# 晚期非小细胞肺癌初始治疗后再次应用EGFR-TKI的疗效观察

**DOI:** 10.3779/j.issn.1009-3419.2011.03.22

**Published:** 2011-03-20

**Authors:** 彤同 安, 真 黄, 玉艳 王, 志杰 王, 桦 白, 洁 王

**Affiliations:** 1 100142 北京，北京大学肿瘤医院胸部肿瘤内科 Department of Thoracic Oncology, Beijing Cancer Hospital, Beijing University Oncology College, Beijing 100142, China; 2 100036 北京，北京积水潭医院骨肿瘤科 Department of Osteoma, Beijing Ji Shui Tan Hospital, Beijing 100036, China

**Keywords:** 肺肿瘤, 表皮生长因子受体, 吉非替尼, 厄洛替尼, Lung neoplasms, Fctor receptor tyrosine kinase inhibitor, Gefitinib, Erlotinib

## Abstract

**背景与目的:**

表皮生长因子受体酪氨酸激酶抑制剂（epidermal growth factor receptor tyrosine kinase inhibitor, EGFR-TKI）是化疗失败的晚期非小细胞肺癌（non-small cell lung cancer, NSCLC）的标准二三线治疗方案，亦是EGFR突变患者一线治疗的最佳选择，但对初始治疗后进展的患者，在治疗过程中能否再次使用TKI是目前的关注热点。本研究旨在探讨晚期NSCLC初始治疗后再次应用EGFR-TKI的疗效。

**方法:**

本研究回顾性分析了71例晚期或术后复发的NSCLC初始EGFR-TKI治疗后，再次应用TKI的疗效。

**结果:**

71例再次应用TKI的患者中，部分缓解（partial response, PR）为7%，稳定（stable disease, SD）为36.6%，（progressive disease, PD）为56.3%，疾病控制率（disease contral rate, DCR）为43.7%，无进展生存期（progression free survival, PFS） > 3个月者26例（36.6%）。EGFR 21外显子突变、初始TKI缓解期≥6个月、两次TKI的间隔期≥3个月与更长的PFS相关，单因素*COX*回归分析，*P*值分别为0.034、0.013、0.046。

**结论:**

TKI治疗失败的NSCLC患者再次应用TKI，部分患者仍可获得疾病控制。EGFR 21外显子突变、初始TKI缓解期≥6个月、两次TKI间隔期≥3个月的患者更可能从再次应用TKI中获益。

## Abstract (en)

**Background and objective:**

The epidermal growth factor receptor tyrosine kinase inhibitor (EGFR-TKI) shows favorable antitumor activity against chemorefractory non-small cell lung cancer (NSCLC). However, patients with advanced NSCLC have limited treatment options available if they are refractory to EGFR-TKI. To study the influence of the retreatment EGFR-TKI after failure of first-line TKI, we carried out this retrospective study.

**Methods:**

Total 71 patients were analyzed who experienced treatment failure from their initial use of EGFR-TKI. After a period of time, they were retreated with TKI as tumor progression was observed.

**Results:**

Of the 71 patients who received retreatment TKI, it was observed in 7% in partial response (PR), 36.6% in stable disease (SD), 56.3% in progressive disease (PD). Disease control rate (DCR) was 43.7%. Twenty-six (36.6%) patients were well controlled by retreatment with TKI monotherapy for not less than 3 months. Five (7.0%) patients had partial response. Exon 21 mutation, PFS not less than 6 months during initial treatment TKI, and the interval not less than 3 months between initial treatment, and retreatment with TKI was associated with a good progression free survival based on univariate *COX* analysis (*P*=0.034; *P*=0.013; *P*=0.046).

**Conclusion:**

It has been shown the possibility that retreatment with TKI might be useful when (1) Exon 21 has active mutation, (2) initial treatment shows a favorable PFS (≥ 6 months), and (3) there has been a period of time (≥3 months) following the termination of the initial TKI treatment.

表皮生长因子受体酪氨酸激酶抑制（epidermal growth factor receptor tyrosine kinase inhibitor, EGFR-TKI）已经成为晚期非小细胞肺癌（non-small cell lung cancer, NSCLC）不可或缺的治疗方法。基于ISEL和BR21等前瞻性临床研究结果^[1, 2]^，EGFR-TKI被推荐为中国晚期NSCLC化疗失败后的标准二三线治疗方案^[3, 4]^；而近年来系列有关一线标准化疗与EGFR-TKI（吉非替尼、厄洛替尼）头对头比较的Ⅲ期前瞻、多中心随机临床研究证明在EGFR突变的患者，一线EGFR-TKI治疗的疗效和无病进展生存时间显著优于化疗^[5, 6]^。*EGFR*突变是EGFR-TKI疗效的强有力预测因子^[6, 7]^，而*KRAS*突变被认为是EGFR-TKI治疗的不敏感的指标，尽管其循证医学证据不如EGFR突变充分^[8, 9]^。

在非选择性患者人群中，EGFR-TKI有效率约为30%-35%，选择性患者（*EGFR*突变者）有效率可达70%-80%^[5]^。初始治疗有效的患者，治疗过程中无一例外均面临因耐药而致的治疗失败。对此部分患者，尤其EGFR-TKI二三线治疗失败或体力状况（phsical status, PS）评分差的患者，目前尚无标准的治疗方案。在治程中能否再次使用EGFR-TKI，及其最佳的应用时间点是目前的关注热点。本研究回顾性分析了在TKI初始治疗NSCLC失败后，再次应用TKI的临床治疗意义。

## 1 材料与方法

### 1.1 病例收集

本研究回顾性的分析了2005年6月-2010年9月，在北京肿瘤医院胸内科接受治疗的71例经病理或细胞学确诊为晚期（Ⅲb期、Ⅳ期）或术后转移复发的NSCLC患者，经初始TKI（吉非替尼、厄洛替尼）治疗后疾病进展，再次使用TKI治疗的情况。其中11例（15.5%）患者TKI作为一线治疗，其余为二三线治疗。所有患者均在随后接受了再次TKI治疗。71例患者中，吉非替尼治疗后再次应用厄洛替尼者56例（78.9%），吉非替尼治疗后再次应用吉非替尼者9例（12.7%），厄洛替尼治疗后再次应用吉非替尼者5例（7.0%），厄洛替尼治疗后再次应用厄洛替尼者1例（1.4%）。

无论初始治疗，还是再次应用，吉非替尼的用法均为250 mg/d，厄洛替尼用法为150 mg/d。疗效评价依据RECIST标准，无进展生存期（progression-free-survival, PFS）定义为从开始治疗至疾病进展。对于TKI疗效的评价，除服药第一个月评效外，此后每两个月进行一次评效。

### 1.2 患者特征

全组患者临床病理特征包括性别、年龄、细胞类型、吸烟状况、基因突变、影像学资料（表 1）。不吸烟定义为一生中吸烟数少于100支。确诊日期、既往化疗方案及疗效均已记录。肺癌的分类依据WHO标准。所有患者均有组织或/和细胞学诊断。

**1:** 本研究纳入的患者特征 Characteristics of patients included in this study Gender

Characteristic	*n*	%
Gender		
Female	45	63.4
Male	26	36.6
Age (year)		
Median	61	
Range	32-80	
Histology		
Squamous cell carcinoma	2	2.8
Adenocarcinoma	68	95.8
Large cell carcinoma	1	1.4
Smoking history		
Smoker	24	33.8
Never	47	66.2
*EGFR* mutation		
Exon 19	27	38.0
Exon 21	13	18.3
Exon 19 and Exon 21	5	7.0
Not available	9	12.7
*KRAS* mutation		
(+)	4	5.6
(-)	28	39.4
Not available	39	54.9
Total enrolled	71	

### 1.3 *EGFR*及*KRAS*突变分析

在71例患者中，62例有组织标本者并进行了*EGFR*突变分析，32例进行了*KRAS*突变分析。肿瘤标本来源包括：手术标本、活检标本、胸水细胞学标本。EGFR19、21外显子突变和*KRAS*突变的检测方法为变性高效液相色谱法（denaturing high performance liquid chromatography, DHPLC）^[9]^。突变的检测均于初次应用TKI前完成。

### 1.4 统计学分析

采用SPSS软件进行统计分析，应用卡方检验比较缓解率和疾病控制率，采用单因素及多因素*COX*回归评价患者基线特征与预后之间的关系。

## 2 结果

### 2.1 患者治疗情况

初始与再次应用EGFR-TKI的中位间隔时间为3个月。11例患者应用TKI作为一线治疗，43例患者于再次TKI治疗前接受化疗，中位化疗周期数为2，方案主要包括吉西他滨+顺铂（卡铂），培美曲塞+顺铂（卡铂），吉西他滨、多西紫杉醇、培美曲塞单药等。19例患者在初始TKI进展后，立即接受二次TKI治疗。

### 2.2 初始TKI治疗患者*EGFR*突变状况与疗效分析

初始TKI治疗的患者中，部分缓解（partial response, PR）为36.6%，稳定（stable disease, SD）为40.8%，疾病进展（progressive disease, PD）为22.5%，疾病控制率（disease contral rate, DCR）为77.4%（表 2）。表 3显示了初始应用TKI的有效率与*EGFR*突变的关系。35例患者存在EGFR突变，初始应用TKI的疗效：PR为54.3%，SD为31.4%，PD为14.3%，DCR为85.7%。27例患者无EGFR敏感突变，初始应用TKI的疗效：PR为22.2%，SD为44.4%，PD为33.3%，DCR为66.6%。初始应用TKI的缓解率在EGFR突变阴性和阳性的患者中差别明显（54.3% *vs* 22.2%, *P*=0.011），DCR两者无明显差别（85.7% *vs* 66.6%, *P*=0.124）。

**2:** 初始及再次应用TKI的疗效 Response to initial and retreatment EGFR-TKI

Response	Initial [*n* (%)]	Retreatment [*n* (%)]
PR	26 (36.6)	5 (7.0)
SD	29 (40.8)	26 (36.6)
PD	16 (22.5)	40 (56.3)
DCR	(77.4)	(43.7)
EGFR-TKI: epidermal growth factor receptor tyrosine kinase inhibitor; PR: partial resonse; SD: stable disease; PD: progressive disease; DCR: disease control rate.		

**3:** 初始应用TKI的疗效与EGFR突变的关系 Relationship between initial treatment of TKI and EGFR mutation

Response	*EGFR* mutation (+) [*n* (%)]	*EGFR* mutation (-) [*n* (%)]
PR	19 (54.3)	6 (22.2)
SD	11 (31.4)	12 (44.4)
PD	5 (14.3)	9 (33.3)
DCR	(85.7)	(66.6)

### 2.3 再次应用TKI治疗患者*EGFR*突变状况与疗效分析

再次应用TKI的患者中，PR为7%，SD为36.6%，PD为56.3%，DCR为43.7%（表 2）。表 4显示了再次应用TKI的有效率与*EGFR*突变的关系。35例患者存在*EGFR*突变，再次应用TKI的疗效：PR为5.7%，SD为45.7%，PD为48.6%，DCR为51.4%。27例患者无*EGFR*突变，再次应用TKI的疗效：PR为11.1 %，SD为44.4%，PD为44.4%，DCR为55.5%。再次应用TKI的缓解率及DCR在*EGFR*突变阴性和阳性的患者中均无明显差别（5.7% *vs* 11.1%, *P*=0.788; 51.4% *vs* 55.5%, *P*=0.849）。

**4:** 再次应用TKI的疗效与EGFR突变的关系 Relationship between retreatment of TKI and EGFR mutation

Response	*EGFR* mutation (+) [*n* (%)]	*EGFR* mutation (-) [*n* (%)]
PR	2 (5.7)	3 (11.1)
SD	16 (45.7)	12 (44.4)
PD	17 (48.6)	12 (44.4)
DCR	(51.4)	(55.5)

43例患者于再次TKI治疗前接受了化疗，28例患者未接受化疗。两组患者再次应用TKI的DCR分别为53.5%和39.3%，无统计学差异（*P*=0.286）。两次TKI间隔时间按是否≥3个月分为两组，间隔时间 < 3个月的患者33例，间隔时间≥3个月的患者38例，DCR分别为33.3%（11例）和65.8%（25例），两者比较有统计学差异（*P*=0.006）。

### 2.4 再次应用TKI的PFS

全组中位PFS为2个月，其中PFS > 3个月的患者26例（36.6%）。比较性别、病理类型、吸烟史、*EGFR*突变及*KRAS*突变的状况，以及初始应用TKI的疗效及缓解期，两次TKI的间隔，间隔期是否应用化疗等因素对PFS的影响，我们发现，在多因素，上述各项均无意义。通过单因素*COX*回归分析GFR 21外显子突变、初始TKI缓解期≥6个月、间隔期≥3个月的患者具有更长的PFS（图 1），为0.034、0.013和0.046。

**1:**
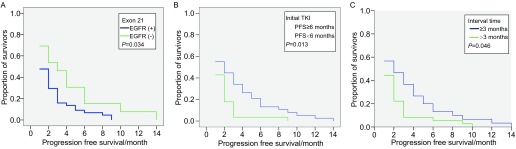
EGFR 21外显子突变（A）、初始TKI缓解期≥6个月（B）及两次TKI间隔期≥3个月（C）对再次应用TKI的影响 Influence of Exon 21 mutation (A), progression free survival not less than 6 months (B), the period of time (not less than 3 months) following the termination of the initial TKI treatment (C) to retreatment TKI

## 3 讨论

EGFR-TKI治疗失败后的后续治疗选择是目前的焦点与热点。本研究回顾性分析71例初始EGFR-TKI治疗后，再次应用EGFR-TKI的临床结果，发现43.6%的患者再次应用TKI仍可获得疾病控制，其中PR为7%，SD为36.6%，中位PFS为2个月。PFS > 3个月的患者达到36.6%。再次应用TKI的有效率和DCR在*EGFR*突变或野生型的患者中未见差异。两次TKI间隔≥3个月的患者与 < 3个月的患者比较，再次应用TKI具有更好的疾病控制率和更长的无进展生存期。

既往有关EGFR-TKI重复治疗的文献^[10-12]^多为回顾性、小样本研究（1例-23例）。近期Kyoichi等^[13]^对11组研究共106例吉非替尼失败后厄洛替尼治疗的患者进行荟萃分析，厄洛替尼治疗的PR、SD、DCR分别为9.9%，18.9%和28.8%，有效率高于我们的报道，但DCR较低。我们和Kyoichi等的研究均显示EGFR-TKI重复治疗至少有约30%-40%患者临床受益。接近于BR21试验的DCR和PFS（分别为45.0%和2.2个月）。

既往研究无一例外均为初始吉非替尼治疗后再次应用厄洛替尼。本研究中吉非替尼治疗后再次应用厄洛替尼者仍占多数（78.9%），但也入组了部分吉非替尼治疗后应用吉非替尼（12.7%），厄洛替尼治疗后应用吉非替尼（7.0%）和厄洛替尼治疗后应用厄洛替尼的患者（1.4%）。对吉非替尼失败后再次应用厄洛替尼获益的解释可能是厄洛替尼血药浓度显著高于吉非替尼^[14]^，且临床前研究显示厄洛替尼对部分野生状态的细胞亦有一定的抑制作用^[15, 16]^。但我们研究中5例PR者，1例来自于吉非替尼治疗失败后再用吉非替尼的患者。34例疾病得到控制的患者中，有6例来自于非吉非替尼后厄洛替尼的治疗组合，因此仅仅用两种药物血药浓度的差异及厄洛替尼对野生状态EGFR细胞的作用更强，不足以圆满解释EGFR-TKI重复应用有效的机制。本研究大部分病例在两次TKI治疗之间均接受了化疗，化疗对*EGFR*突变状态有无影响目前尚有争议，我们的研究显示化疗后*EGFR*突变发生了变化，总的趋势为突变比例下降。化疗前后70%的患者*EGFR*突变状态保持不变，30%由阴性转变成阳性或由阳性转变成阴性（文章待发表），此种转变是否是导致EGFR-TKI重复应用有效的机制之一尚需深入研究。

本研究62例患者进行了*EGFR*基因的检测，我们发现EGFR 21外显子突变与野生型比较，再次应用TKI有更长的PFS。而以往认为Exon 19突变与TKI的疗效有更强的相关性。我们还发现，初始TKI缓解期≥6个月，再次应用TKI者有更长的PFS，这和以往的类似报道^[10-12]^相同。本研究首次报道两次TKI间隔≥3个月的患者再次应用TKI具有更长的PFS。对于TKI耐药的机制目前尚不清楚。耐药基因的变化（包括T790M、c-MET）对重复应用EGFR-TKI有无影响目前尚不明确。我们推测，如果患者存在T790M、c-MET变异，再次应用TKI可能无效。

本研究为回顾性分析，目前的类似研究均停留在个案报道和小样本的回顾性分析上，因此我们期待更多的大样本前瞻性研究。
